# PPARγ Activates Autophagy by Suppressing the PI3K–AKT1–FOXO3 Signaling Pathway and thus Alleviates Hepatic Ischemia-Reperfusion Injury

**DOI:** 10.5152/tjg.2025.24529

**Published:** 2025-04-21

**Authors:** Xinyu Liu, Hengguan Cui, Xianqing Song, Weixing Shen, Bin Yan

**Affiliations:** 1 Department of General Surgery, Qingpu Branch of Zhongshan Hospital Affiliated to Fudan University, Shanghai, China; 2 Department of General Surgery, Baoan Central Hospital, Shenzhen, China

**Keywords:** Autophagy, hepatic ischemia-reperfusion injury, PI3K–AKT1–FOXO3, PPARγ

## Abstract

**Background/Aims:**

Hepatic ischemia-reperfusion injury (HIRI) refers to the damage caused by metabolic imbalance post-ischemia upon reperfusion, often occurring in scenarios like hemorrhagic shock, liver resection, and liver transplantation. Due to the complex nature of the mechanisms underlying metabolic imbalance, specific treatment options are lacking. Peroxisome proliferator activated receptor gamma (PPARγ) is a group of metabolic regulatory receptors that can influence HIRI by regulating autophagy, although the precise mechanism remains contentious.

**Materials and Methods:**

In vivo and in vitro experiments were conducted to simulate hypoxic conditions, evaluating the effects of PPARγ overexpression plasmids, autophagy inhibitors, phosphatidylinositol 3-kinase (PI3K) activators, and PPARγ agonists on HIRI. The activation status of the PI3K–AKT1–FOXO3 signaling pathway, autophagy levels, inflammatory responses, and liver cell/organ damage were analyzed using western blot, ELISA, flow cytometry, H&E staining, and TUNEL experiments.

**Results:**

Peroxisome proliferator activated receptor gamma can mitigate cell damage caused by hypoxia by activating autophagy, with the activation of autophagy being associated with the inhibition of the PI3K–AKT1–FOXO3 signaling pathway. Additionally, pretreatment of mice with the PPARγ agonist rosiglitazone can alleviate HIRI induced by ischemia by inhibiting the activation of the PI3K–AKT1–FOXO3 signaling pathway to induce autophagy.

**Conclusion:**

Peroxisome proliferator activated receptor gamma inhibited the PI3K-AKT1-FOXO3 signaling pathway, which in turn activated autophagy to alleviate HIRI.

**Main Points**

- Peroxisome proliferator activated receptor gamma (PPARγ) can mitigate cell damage caused by hypoxia by activating autophagy.
- The activation of autophagy is associated with the inhibition of the PI3K-AKT1-FOXO3 signaling pathway.
- The pretreatment of mice with the PPARγ agonist rosiglitazone can alleviate HIRI induced by ischemia by inhibiting the activation of the PI3K–AKT1–FOXO3 signaling pathway to induce autophagy.

## Introduction

The damage brought about by a metabolic imbalance following ischemia and subsequent reperfusion is known as hepatic ischemia-reperfusion injury (HIRI). This phenomenon frequently affects patients who have had liver resection, liver transplantation, or hemorrhagic shock. It can cause immunological rejection, liver failure, and dysfunction, all of which have a major negative influence on the prognosis of these patients.^1^^,^^2^ Research has revealed that HIRI is mostly caused by anaerobic metabolism, mitochondrial damage, oxidative stress, reactive oxygen species production, Ca^2+^ ion excess, chemokines, and nitric oxide .^3^ Nonetheless, there are still a lot of pathways that are unknown, which somewhat restricts how HIRI patients can be treated.

One of the most researched members of the nuclear receptor superfamily, peroxisome proliferator activated receptor gamma (PPARγ) binds to a variety of ligands, such as fatty acids and prostaglandins, to regulate endothelial function, oxidative stress, inflammation, and glucose/lipid metabolism.^4^ As a potential therapeutic target, PPARγ has demonstrated potential benefits in treating several illnesses, including osteoarthritis,^5^ non-alcoholic fatty liver disease,^6^ type 2 diabetes,^7^ and allergy disorders.^8^ It is worth noting that the PPARγ agonist rosiglitazone also has a protective effect on HIRI mice.^9^ However, the exact mechanism of this protection remains controversial. On one hand, the activation of PPARγ is associated with a reduction in the formation and clearance of autophagosomes.^10^ On the other hand, high expression of PPARγ is accompanied by low expression of autophagy-related proteins.^11^ Autophagy is a two-edged sword that plays a role in both cellular damage under environmental stress and in maintaining cellular and organismal homeostasis through the degradation and recycling of proteins and organelles.^12^ This process is closely related to developing various metabolism-related diseases.^13^ Therefore, a thorough exploration of the regulatory role of the PPARγ/phosphatidylinositol 3-kinase (PI3K) signaling pathway on autophagy can not only explain the mechanism of HIRI but also provide crucial evidence for the application of PPARγ targets.

The work aimed to validate the alterations in autophagy levels during PPARγ regulation of HIRI to tackle this issue. Moreover, it explored how autophagy regulation by the PI3K–AKT1–FOXO3 axis affected HIRI. The results of this study clarified the processes by which PPARγ controlled HIRI and offered fresh treatment avenues for HIRI.

## Materials and Methods

### Cell Lines and Culture Conditions

The source of the mouse normal liver cells (AML12) was Wuhan Pricella Biotechnology Co., Ltd. (CL-0602, China). The selection of target cells refers to previous studies.^14^^,^^15^ The cells were cultivated in 10% fetal bovine serum (FBS) (164210, Procell, China), 0.5% ITS-G (PB180429, Procell, China), 40 ng/mL dexamethasone (ST1254, Beyotime, China), and 1% penicillin–streptomycin (PB180120, Procell, China) supplemented in DMEM/F12 medium (PM150312, Procell, China). The cells were passaged and cultivated in a humidified environment with 5% CO_2_ at 37°C.

### Transfection

The oe-NC and oe-PPARγ plasmids were acquired from GenScript Biotech Corporation (China). As directed by the manufacturer of Lipofectamine^TM^ 2000 transfection reagent (11668019, Thermo Fisher Scientific, USA), transfection was performed. In short, cells were seeded in 6-well plates to grow to 70% confluency. Opti-MEM medium (31985070, Thermo Fisher Scientific, USA) was used in place of the culture media. After pre-mixing the transfection reagent with plasmids, they were introduced to the cells dropwise. The media was switched to a full culture medium for the ensuing studies after 6 hours.

### Cell Grouping and Hypoxia/Reoxygenation (H/R) Treatment

Cells in the control group were cultured normally. The cells in other groups were transfected with oe-NC/oe-PPARγ plasmid and treated with hypoxia 24 hours later. To mimic hypoxic conditions, cells in the treatment groups were briefly exposed to Earle’s balanced salt solution (PB180337, Procell, China) containing 0.3 mM Na_2_S_2_O_4_ (S817915, Macklin, China). After 4 hours, the media was switched to a full culture medium supplemented with DMSO, autophagy inhibitor (10 μM Chloroquine, CQ) (HY-17589A, MedChemExpress, USA), and PI3K activator (30 μM 740 Y-P) (HY-P0175, MedChemExpress, USA) for reoxygenation for 12 hours. The H/R treatment conditions were referenced from previous studies and optimized.^15^^,^^16^

### RNA Extraction and Reverse Transcription Quantitative Real-Time Polymerase Chain Reaction

With the use of TRIzol reagent (15596018CN, Invitrogen, USA), total RNA from the cells was isolated. Then, cDNA was generated using the Hifair^®^ II first strand cDNA synthesis kit (11119ES60, YESEN, China). Real-time quantitative PCR (qPCR) analysis was carried out using uGreener Flex qPCR 2X Mix (Q0005A, U&G BIO, China) on an Applied Biosystems™ 7500 qRT-PCR system (4351105, Invitrogen, USA). Using the 2^-ΔΔCT^ technique and β-actin as the reference gene, the relative expression of PPARγ mRNA in the cells was ascertained. Primer sequences were referenced from a previous study,^17^ as shown in Table 1. The qPCR program settings: 95°C for 30 s; 95°C for 5 s, and 60°C for 30 s for 40 cycles; then held at 4°C.

### Construction and Treatment of Hepatic Ischemia-Reperfusion Injury Mouse Model

Ten Balb/c mice, aged 6-8 weeks (N000020, GemPharmatech, China), were split into 2 groups of 5 mice each at random. Following acclimation to the laboratory setting, 1 group underwent daily intraperitoneal injections of rosiglitazone (1 mg/kg) (HY-17386, MedChemExpress, USA), while the other group underwent weekly injections of 10% DMSO (2 mL/kg) (HY-Y0320, MedChemExpress, USA).

Mice were given isoflurane anesthesia (792632, Merck, Germany) after a week. The liver hilum was completely visible once the abdominal cavity was exposed. To cause hepatic ischemia, a microvascular clamp was used to clamp the left hepatic vein for 1 hour. After that, the clamp was taken out, the abdominal cavity was sealed, and blood flow was resumed for 6 hours. The construction of the HIRI mouse model was based on previous research.^18^

The animal study protocol has been approved by the Animal Ethics Committee of Guangdong Medical Laboratory Animal Center, with approval number D202411-9 on November 27, 2024. This study adhered to the guidelines established by the committee.

### Enzyme-Linked Immunosorbent Assay

After collecting cell culture supernatants from every treatment group, the levels of interleukin-6 (IL-6) and tumor necrosis factor-alpha (TNF-α) were determined using mouse TNF-α and IL-6 enzyme-linked immunosorbent assay (ELISA) kits (ab208348/ab222503, Abcam, UK).

Each mouse had its whole blood sample taken and left to stand at room temperature for an hour, and then samples were centrifuged at 1500 g and 4°C for 5 minutes to extract serum. The TNF-α and IL-6 were assayed as described above. Using alanine aminotransferase (ALT) and aspartate aminotransferase (AST) test kits (ab282882/ab263882, Abcam, UK), the quantities of ALT and AST in the serum were determined.

### Western Blot Analysis

Total proteins from lung adenocarcinoma cells and tissues were extracted for WB analysis using ice-cold RIPA buffer (P0013B, Beyotime, China) containing 1% protease inhibitors and PMSF (ST506, Beyotime, China). After 10 minutes of lysis, the samples were centrifuged at 12 000 rpm for 15 minutes. The BCA protein assay kit (PC0020, Solarbio, China) was used to measure the protein content of the samples. sodium dodecyl sulfate-polyacrylamide gel electrophoresis was used to separate the proteins, which were then transferred to a polyvinylidene fluoride membrane. After adding LC3B, Beclin 1, AKT1, Phospho-AKT1-S473, FOXO3A and Phospho-FOXO3A-S253 Rabbit pAb primary antibodies (A11282/A7353/A11016/AP0140/A0102/AP0684, Abclonal, USA), the appropriate HRP-conjugated Goat anti-Rabbit IgG (H + L) secondary antibodies (AS014, Abclonal, USA) were added. Using the ChemiScope 6000 imaging equipment (Clinx, China), pictures were taken following treatment with ECL substrate (BL520A, Biosharp, China).

### Flow Cytometry Analysis

Cell apoptosis was examined using flow cytometry. The manufacturer’s instructions were followed to digest the fibroblasts with 0.25% trypsin without EDTA (C0201, Beyotime, China), wash the cells twice in PBS, and label the cells using the Annexin V-FITC/PI apoptosis detection kit (AP101, MULTI SCIENCES, China). To attain a density of 1 × 10^6^ cells/mL, cell suspensions were produced in 1× Binding buffer. About 500 μL of suspensions were added with 5 μL Annexin V-FITC and 10 μL PI, mixed, and incubated for 5 minutes at room temperature in the dark. The NovoCyte flow cytometry system (Agilent, USA) was used for detection.

### Hematoxylin and Eosin Staining

After being dried and mounted, liver tissues from mice that were euthanized were preserved in 4% paraformaldehyde (P0099, Beyotime, China) overnight. The tissues were then dehydrated, paraffin-embedded, and sectioned using a microtome. Sections were deparaffinized, hydrated, and stained with hematoxylin (C0107, Beyotime, China) and eosin (C0109, Beyotime, China). After dehydration and encapsulation, a photographic record was made.

### Terminal deoxynucleotidyl transferase dUTP Nick-End Labeling (TUNEL) Assay

A 1-step TUNEL apoptosis detection kit (C1088, Beyotime, China) was used to perform the TUNEL experiment on liver tissue paraffin slices. Using xylene, deparaffinization was accomplished. Gradient ethanol and PBS solution were then applied. Sections were treated with 20 μg/mL proteinase K (ST532, Beyotime, China) free of DNase, rinsed with PBS, stained with TUNEL detection solution, and then incubated for 60 minutes at 37°C in the dark. They were counterstained with DAPI (C1005, Beyotime, China) for 5 minutes, rinsed with PBS, mounted with anti-fade mounting material (HY-K1042, MedChemExpress, USA), and examined and photographed under a fluorescence microscope.

### Statistical Analysis

Every experiment was conducted 3 times, and GraphPad Prism 8.3.0 (GraphPad Software; San Diego, USA) was used for analysis. The data are shown as mean ± SD. Student’s *t*-test and 2-way ANOVA were used to assess differences, with *P* < .05 regarded as statistically significant.

## Results

### Peroxisome Proliferator Activated Receptor Gamma Alleviates Cell Damage Induced by Hypoxia/Reoxygenation Through Activating Autophagy

**Figure 1.**
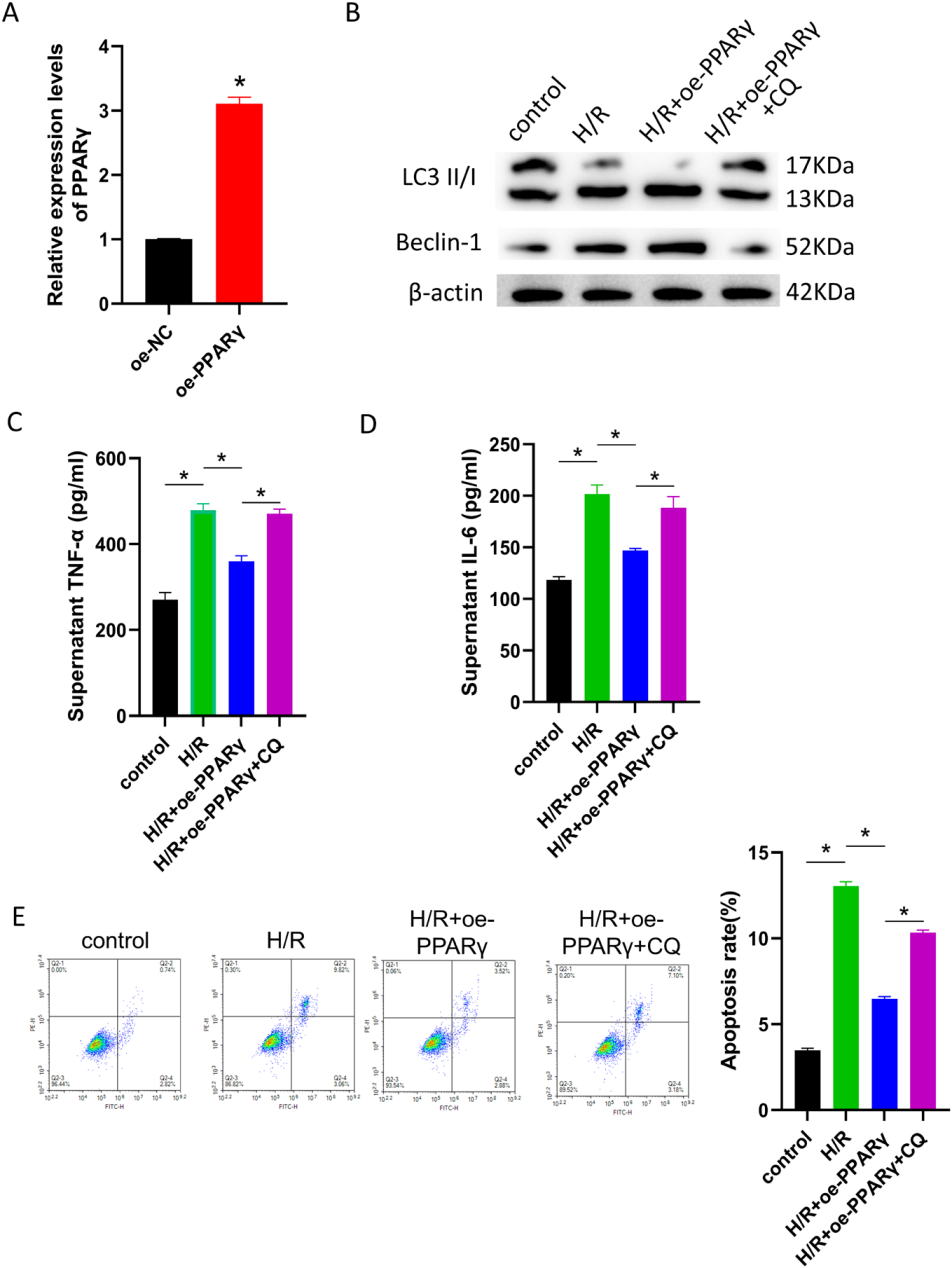
PPARγ alleviates cell damage induced by H/R through activating autophagy. The oe-PPARγ plasmid was transfected into AML12 cells. Then H/R treatment was performed, and CQ was added to regulate autophagy levels. (A) qRT-PCR analysis of PPARγ expression in cells; (B) WB analysis of autophagy-related proteins (LC3 II/I and Beclin-1) expression in cells; (C) ELISA measurement of TNF-α (inflammatory factor) levels in cell culture supernatants; (D) ELISA measurement of IL-6 (inflammatory factor) levels in cell culture supernatants; and (E) flow cytometry analysis of apoptotic cell proportions (Q2-2 region). *Represents *P* < .05, indicating statistical significance.

The PPARγ overexpression plasmid oe-PPARγ and its control oe-NC were generated to explore the regulatory function of PPARγ in HIRI. Two sets of cells with varied levels of PPARγ expression were developed following the transfection of AML12 cells (Figure 1A). The cells were then sorted and exposed to H/R therapy. During reoxygenation, an autophagy inhibitor (CQ) was introduced to disrupt the autophagic process. The expression of autophagy-related proteins LC3 II/I and Beclin-1 was shown to be elevated during H/R treatment, according to WB data. Additionally, the overexpression of PPARγ further triggered autophagy, which was suppressed by the addition of CQ (Figure 1B). In addition, measurements were made of the inflammatory markers TNF-α and IL-6 in cell culture supernatants. The H/R treatment exacerbated the inflammatory response of cells in each group, and overexpression of PPARγ partially alleviated this situation. The inflammatory reaction was made worse again by the addition of CQ (Figure 1C and D). Lastly, flow cytometry was used to measure apoptosis in each set of cells. Peroxisome proliferator activated receptor gamma overexpression attenuated the considerable increase in the fraction of apoptotic cells caused by H/R therapy. On the other hand, apoptotic cell proportions increased again when CQ was added (Figure 1E). These experimental results imply that PPARγ can prevent H/R-induced cell damage by inducing autophagy.

### Peroxisome Proliferator Activated Receptor Gamma Attenuates Cell Damage Induced by Hypoxia/Reoxygenation Through Inhibition of the PI3K/AKT1/FOXO3 Signaling Pathway to Activate Autophagy

**Figure 2.**
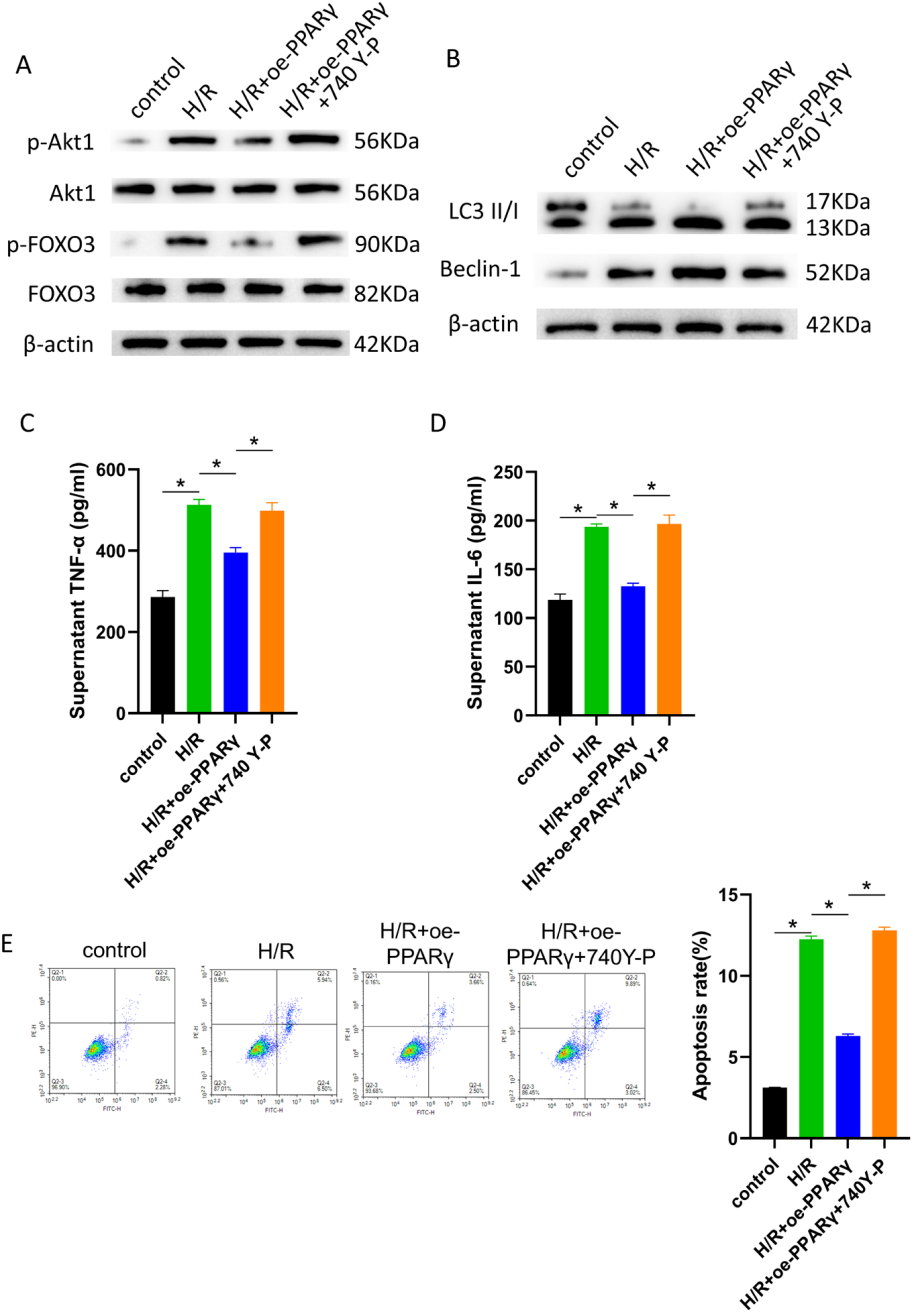
PPARγ attenuates cell damage induced by H/R through inhibition of the PI3K/AKT1/FOXO3 signaling pathway to activate autophagy. The oe-PPARγ plasmid was transfected into AML12 cells. Then H/R treatment was performed, and 740 Y-P was added to regulate the PI3K signaling pathway. (A) WB analysis of PI3K signaling pathway proteins (AKT1, p-AKT1, FOXO3, and p-FOXO3) expression; (B) WB analysis of autophagy-related proteins (LC3 II/I and Beclin-1) expression; (C) ELISA measurement of TNF-α (inflammatory factor) levels in cell culture supernatants; (D) ELISA measurement of IL-6 (inflammatory factor) levels in cell culture supernatants; (E) Flow cytometry analysis of apoptotic cell proportions (Q2-2 region). *Represents *P* < .05, indicating statistical significance.

This work concentrated on the upstream signaling pathway PI3K/AKT1/FOXO3,^19^ which may affect autophagy, to learn more about the processes by which PPARγ regulates autophagy to affect cell damage. A combination of a PI3K agonist (740 Y-P) was used to treat cells from each group to confirm the link between this signaling pathway and cell damage. The findings of WB analysis showed that following H/R therapy, the phosphorylation levels of AKT1 and FOXO3 increased, but these were suppressed by PPARγ overexpression. The PI3K/AKT1/FOXO3 signaling pathway was reactivated by using a PI3K agonist (Figure 2A). This suggests that H/R therapy triggered the system, which was repressed by the overexpression of PPARγ, preventing signal transduction via this route. The expression of autophagy-related proteins LC3 II/I and Beclin-1 was raised following H/R treatment and further activated by overexpressed PPARγ. This process was repressed with the use of the PI3K agonist (Figure 2B), illustrating that PPARγ may target the PI3K/AKT1/FOXO3 pathway to promote cell autophagy in the H/R model. Furthermore, the findings of ELISA demonstrated a noteworthy rise in TNF-α and IL-6 levels in the cell culture supernatant of the H/R cell model. These increases were impeded by the overexpression of PPARγ, but this tendency was reversible with the activation of the PI3K pathway (Figure 2C and D). The overexpression of PPARγ reduced the considerable increase in cell apoptosis seen by flow cytometry following H/R therapy. Cell apoptosis levels, however, increased again after the PI3K agonist was added (Figure 2E). These findings indicate that PPARγ can reduce the damage to cells caused by H/R by suppressing the PI3K/AKT1/FOXO3 signaling pathway, which triggers autophagy.

### Pretreatment with Peroxisome Proliferator Activated Receptor Gamma Agonist (Rosiglitazone) Alleviates Hepatic Ischemia-Reperfusion Injury in Mice

**Figure 3.**
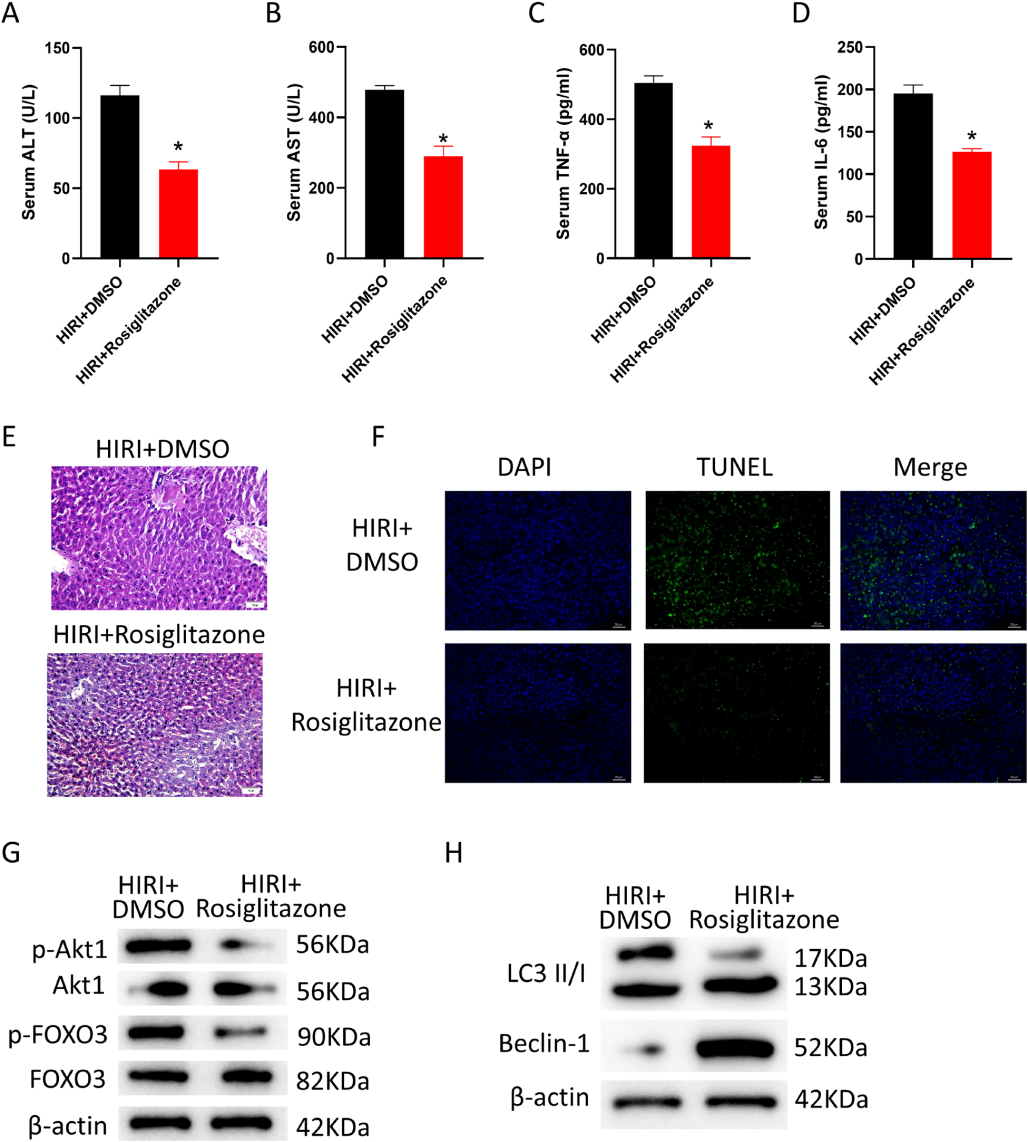
Pretreatment with PPARγ agonist rosiglitazone alleviates HIRI in mice. Mice were pretreated with rosiglitazone to activate PPARγ targets. Subsequently, mice livers were treated with H/R. (A) ELISA measurement of serum ALT (tissue damage indicator) levels; (B) ELISA measurement of serum AST (tissue damage indicator) levels; (C) ELISA measurement of serum TNF-α (inflammatory factor) levels; (D) ELISA measurement of serum IL-6 (inflammatory factor) levels; (E) H&E staining of liver tissue, magnification 200×. The blue-purple color represents the nucleus, and the red color represents the cytoplasm; and (F) TUNEL staining of liver tissue, magnification 200×. Blue fluorescence represents all cells, green fluorescence represents apoptotic cells; (G) WB analysis of PI3K signaling pathway proteins (AKT1, p-AKT1, FOXO3, and p-FOXO3) expression in liver tissue; (H) WB analysis of autophagy-related proteins (LC3 II/I and Beclin-1) expression in liver tissue. *Represents *P* < .05, indicating statistical significance.

In this work, mice that had received rosiglitazone as a pretreatment were given H/R therapy to confirm the crucial regulatory function of PPARγ targets in HIRI. According to the experimental data, mice pretreated with rosiglitazone had considerably lower serum levels of tissue damage indicators (ALT, AST) compared to the control group (Figure 3A and B), as well as lower levels of inflammatory markers (TNF-α, IL-6) (Figure 3C and D). Furthermore, mice given rosiglitazone had less necrotic liver tissue than the control group, according to H&E staining data (Figure 3E). Accordingly, the TUNEL assay findings showed that mice given rosiglitazone had a lower apoptotic ratio of liver cells than the control group (Figure 3F). The expression levels of Beclin-1, LC3 II/I, FOXO3, p-FOXO3, p-AKT1, and AKT1 in liver tissues were then assessed. According to WB analysis, pretreatment with rosiglitazone decreased the phosphorylation of AKT1 and FOXO3 in comparison to the control group (Figure 3G) while simultaneously upregulating the expression of proteins linked to autophagy (LC3 II/I, Beclin-1) (Figure 3H). These results from the experiments indicate that rosiglitazone pretreatment reduces HIRI in mice, a mechanism that is closely related to autophagy activation mediated by the PI3K/AKT1/FOXO3 axis.

## Discussion

A consequence of liver ischemia, known as HIRI, is characterized by damage mostly brought on by oxidative stress and inflammatory reactions.^20^^-^^22^ The precise mechanisms at play are quite complex.^18^^,^^23^ As a result, supportive care is the focus of current management, with no therapeutic approaches.^24^ PPARγ functions as an essential regulatory target in HIRI, as PPARγ-deficient mice show more severe liver damage after ischemia-reperfusion (IR).^9^ Peroxisome proliferator activated receptor gamma activation may be able to restore damage caused by IR.^25^ The effectiveness of this target in several animal models has been confirmed by a variety of natural and synthetic PPARγ agonists.^26^^,^^27^ Therefore, investigating PPARγ regulatory mechanisms in more detail seems promising for treating HIRI.

In this study, it was found that overexpression of PPARγ can alleviate HIRI by activating autophagy. This result corroborates the research on Yes-associated protein. Yes-associated protein can prevent HIRI by activating autophagy.^28^ Mechanistically, moderate autophagy upregulates the expression of molecular chaperones, thereby enhancing endogenous defense mechanisms.^29^ At the same time, it was demonstrated that PPARγ primarily activates autophagy by inhibiting the PI3K signaling pathway. This regulatory relationship has been shown to alleviate oxidative stress-induced damage in osteoblasts.^30^ Therefore, the role of the PI3K-autophagy regulatory axis in the treatment of HIRI should be emphasized.

Notably, changes in autophagy levels are linked to the functional role of PPARγ, albeit the exact processes involved are still up for debate. Ruan et al^10^ have pointed out that preconditioning rats with distant limb ischemia can increase PPARγ expression, trigger autophagy, and relieve HIRI. In contrast, autophagy is suppressed during PPARγ knockdown, which exacerbates HIRI in mice.^17^ However, further studies indicate that HIRI can be reduced by pre-treatment with different drugs, although at the expense of autophagy inhibition.^31^^,^^32^ The lack of a clear correlation between activated autophagy levels and HIRI raises questions about potential applications of the PPARγ targets.

Using PPARγ agonists, PI3K activators, and autophagy inhibitors, as well as PPARγ overexpression plasmids, the effects of these agents were confirmed in vitro and in vivo to further explore this pathway. Results from experiments showed that PPARγ could activate autophagy, reduce inflammatory responses, and inhibit cell apoptosis to lessen the harm that hypoxia causes to cells. These outcomes are consistent with the findings of Wu et al,^17^ who found that PPARγ activates autophagy through the AMPK–mTOR signaling pathway, thereby mitigating hypoxia-induced hepatic damage. In addition, by blocking the NF-κB signaling pathway, PPARγ can reduce inflammation, oxidative stress, cell apoptosis, and liver damage.^25^ This work showed that the inhibition of the PI3K–AKT1–FOXO3 signaling pathway was related to the activation of autophagy mediated by PPARγ. The PI3K–AKT signaling axis is a well-known autophagy-regulating factor,^33^ and the downstream target gene FOXO3 is a crucial player in HIRI.^19^^,^^34^ Autophagy activated by this axis reduces HIRI. Liu et al^35^ unveiled that autophagy was repressed, and the PI3K–AKT signaling pathway was stimulated throughout the process of shikonin relieving HIRI. This emphasizes the intricate regulatory processes behind HIRI, wherein autophagy activation does not always translate into HIRI relief. Significantly, this study showed that pretreatment with the PPARγ agonist rosiglitazone could suppress inflammation and organ damage brought on by IR by inhibiting the PI3K–AKT1–FOXO3 signaling pathway in mice livers and triggering autophagy. This offers solid experimental evidence in favor of rosiglitazone as a treatment for HIRI.

In summary, this work has shown that PPARγ can suppress the PI3K–AKT1–FOXO3 signaling pathway, which in turn activates autophagy to reduce HIRI. These results highlight the effect of PI3K–AKT1–FOXO3 axis-mediated autophagy suppression on HIRI and confirm the use of PPARγ agonists in the treatment of HIRI. Unfortunately, the mechanism by which FOXO3 regulates autophagy in our study remains unclear. Additionally, further quantification is needed to determine the extent to which autophagy can alleviate HIRI. Supplementing these findings will provide an important theoretical basis for the development of PPARγ targets. Later, more investigations will be done to clarify how PPARγ controls HIRI and to develop targeted treatment strategies for HIRI.

## Figures and Tables

**Table 1.** qPCR Primer Sequence

Primers	Sequence (5’-3’)
PPARγ-F	GTCTTGGATGTCCTCGATGGG
PPARγ-R	TTATGGAGCCTAAGTTTGAGTTTGC
β-actin-F	GGCTGTATTCCCCTCCATCG
β-actin-R	CCAGTTGGTAACAATGCCATGT

## Data Availability

The data and materials in the current study are available from the corresponding author upon reasonable request.
